# Polygenic risk scores and brain structures both contribute to externalizing behavior in childhood - A study in the Adolescent Brain and Cognitive Development (ABCD) cohort

**DOI:** 10.1016/j.nsa.2023.101128

**Published:** 2023-05-24

**Authors:** Jalmar Teeuw, Nina Roth Mota, Marieke Klein, Neeltje E. Blankenstein, Jorim J. Tielbeek, Lucres M.C. Jansen, Barbara Franke, Hilleke E. Hulshoff Pol

**Affiliations:** a Department of Psychiatry, Brain Center Rudolf Magnus, University Medical Center Utrecht, Utrecht, the Netherlands; b Donders Institute for Brain, Cognition and Behaviour, Radboud University, Nijmegen, the Netherlands; c Department of Human Genetics, Radboud University Medical Center, Nijmegen, the Netherlands; d Department of Psychiatry, University of California, San Diego, La Jolla, CA, United States; e Department of Child and Adolescent Psychiatry and Psychosocial Care, Amsterdam UMC, Vrije Universiteit Amsterdam, the Netherlands; f Department of Developmental and Educational Psychology, Institute of Psychology, Leiden University, Leiden, the Netherlands; g Department of Psychiatry, Radboud University Medical Center, Nijmegen, the Netherlands; h Department of Experimental Psychology, Helmholtz Institute, Utrecht University, Utrecht, the Netherlands

**Keywords:** Externalizing behaviors, Late childhood, Adolescence, Gray matter volume, White matter integrity, Polygenic risk scores, Magnetic resonance imaging

## Abstract

**Introduction:**

Externalizing behaviors are defined as behaviors violating social norms and can be harmful to self and others. Predicting the escalation of externalizing behaviors in children would allow for early interventions to prevent the occurrence of antisocial and criminal acts. Externalizing behaviors are heritable traits, and have been associated with structures of the brain. Brain structure, in turn, is also influenced by genetics. Here, we investigated the association of genetic and brain structural variation with externalizing behaviors in late childhood, and we assessed potential mediating effects.

**Methods:**

Data was collected for 11,878 children aged 9–10 years old from the Adolescent Brain Cognitive Development (ABCD) cohort. We extracted data on externalizing behaviors measured by the parent-reported Child Behavior Checklist (CBCL), brain volumes and white matter integrity measured by magnetic resonance imaging (MRI), and polygenic risk scores (PRS) for (i) antisocial behaviors, (ii) attention-deficit/hyperactivity disorder comorbid with disruptive behavior disorder (ADHD ​+ ​DBD), (iii) irritability, and (iv) traits related to self-regulation & addiction. We examined the associations between brain structures, PRS, and externalizing behavior, and to what extent brain structures mediate the association between PRS and externalizing behavior. Phenotypic associations between brain structures and externalizing behaviors were validated in an independent cohort of 150 adolescents aged 12–21 years enriched for individuals with antisocial behavior.

**Results:**

Increasing levels of externalizing behaviors were associated with reduced total brain and focal gray matter volumes, but not with white matter integrity. These results could not be validated in the independent cohort, except for a good correlation of several effect sizes between the cohorts. Higher PRS for externalizing behaviors were associated with lower cortical gray matter volume, larger subcortical gray matter volume, larger white matter volume, and reduced global white matter fractional anisotropy. Genetic and brain structural variation, combined with sociodemographic factors, explained up to 7% of variation in externalizing behaviors in late childhood; brain structures and PRS each explained up to ∼0.5% of variation. A multivariate model with all sociodemographic factors, brain structures and PRS combined explained up to 11.9% (+5%). Total cortical gray matter volume mediated the association between PRS for ADHD ​+ ​DBD and externalizing behavior in late childhood. However, a large proportion of individual variation in externalizing behavior remained unidentified (∼90%). Brain function and interaction effects with the environment are surmised as potential sources of additional variation.

## Highlights

• **Externalizing behavior is associated with smaller brain volumes, but not white matter integrity, and could largely be explained by total brain volume.**
• **Polygenic risk scores (PRS) for externalizing-related phenotypes are associated with externalizing behavior in childhood.**
• **PRS for aggressive-related externalizing behaviors are associated with global brain volumes and white matter integrity.**
• **Total gray matter volume mediated the association between the PRS for disruptive behavior in ADHD and externalizing behavior.**
• **PRS for aggressive-related externalizing behaviors and brain structures have a unique contribution to externalizing behavior.**

## 1 Introduction

Externalizing behaviors are characterized as antisocial and disruptive behaviors in the form of aggression, delinquency, or criminal acts that can be costly and potentially harmful to the individual, their family and friends, and society (Christenson et al., 2016). Externalizing behaviors occur most often during childhood and adolescence (Fairchild et al., 2019; Isen et al., 2022; Loeber and Burke, 2011), and they can be predictive of emotional and behavioral problems later in life, including criminal behavior (Erskine et al., 2016; Kjeldsen et al., 2016). Although current evidence points to a genetic contribution to externalizing behaviors, and several brain structures have been associated with externalizing behaviors, the underlying neurobiological pathways remain unresolved.

Twin and family studies have revealed heritable factors for both externalizing behaviors and brain structures (Gard et al., 2019; Isen et al., 2022; Luningham et al., 2020; Polderman et al., 2015; Porsch et al., 2016; van der Valk et al., 2003; Wesseldijk et al., 2018). Recently, genome-wide association studies (GWAS) have identified loci on the human genome associated with aspects of externalizing behaviors in children and adults (Baselmans et al., 2021; Demontis et al., 2021; Ip et al., 2021; Karlsson Linnér et al., 2021; Tielbeek et al., 2017, 2021; Watanabe et al., 2019); similarly, GWAS for brain structure and function across the lifespan, including longitudinal change rates of brain structures, have been conducted (Brouwer et al., 2022; Grasby et al., 2020; Hofer et al., 2020; Jahanshad et al., 2017; Satizabal et al., 2019; Smith et al., 2021; Thompson et al., 2020). Based on GWAS results, polygenic risk scores (PRS) can be computed to determine the genetic risk load for a phenotype carried by each individual in an independent cohort, and these can then be used to investigate the genetic association with related phenotypes (Wray et al., 2021). Previous studies have successfully reported on the association of PRS for neurodevelopmental/psychiatric disorders and cognition with brain structure and function (Albaugh et al., 2019; Fernandez-Cabello et al., 2022). E.g., Albaugh et al. reported that an increased genetic risk for ADHD is associated with decreased fractional anisotropy in the superior and inferior longitudinal fasciculus, and Fernandez-Cabello et al. reported that increased polygenic risk for several psychiatric disorders are associated with alterations in brain structures.

The human brain continues to develop considerably from birth through early adulthood (Blakemore, 2012; Giedd et al., 1999; Gogtay et al., 2004). Many properties of brain structures are highly heritable throughout the lifespan (Blokland et al., 2012; Douet et al., 2014; Jansen et al., 2015; Strike et al., 2015), including their longitudinal change rates during adolescent brain development and alterations in psychiatric disorders (Brans et al., 2008, 2010; Brouwer et al., 2014, 2017; Hedman et al., 2016; Teeuw et al., 2019; van Soelen et al., 2012, 2013). Externalizing behaviors have been linked to alterations in the structure and functioning of the brain (Baker et al., 2015; Blair and Zhang, 2020; Johanson et al., 2019; Noordermeer et al., 2016; Waller et al., 2017). Overall, various forms of externalizing behavior and psychopathology have been associated with reduced gray matter volume in structures of the frontal and temporal cortex and the limbic system (Bayard et al., 2020; Cohn et al., 2016; Durham et al., 2021; Raine et al., 2000; Waller et al., 2020), as well as with increased white matter volume and/or reduced white matter integrity of the brain (Andre et al., 2020; Bolhuis et al., 2019a, 2019b; Neumann et al., 2020; Pape et al., 2015; Raine et al., 2003). Moreover, longitudinal changes in externalizing behaviors have been associated with changes in gray matter (Ameis et al., 2014; Bos et al., 2018; Caldwell et al., 2015; Oostermeijer et al., 2016; Roberts et al., 2021; Tanzer et al., 2021; Whittle et al., 2020) and with changes in white matter structures (Andre et al., 2020; Garic et al., 2019; Jones et al., 2019; Muetzel et al., 2018).

Previously, we have shown in a longitudinal twin cohort that self- and parent-reported externalizing behaviors and brain structures share a genetic factor during adolescent development (Teeuw et al., 2022). However, it remains unknown to what extent the genetic risk load related to externalizing behaviors is associated with the development of brain structures in late childhood, and whether these brain structures mediate the relationship between the genetics of externalizing behaviors and the display of such behaviors. In the current study, we examined the association between parent-reported externalizing behavior measures and brain structures in a large cohort of almost 12 thousand children of 9–10 years old from the Adolescent Brain and Cognitive Development (ABCD) study. The main aim of this study was to investigate the association of brain structural variation and PRS based on externalizing phenotypes with externalizing behaviors in late childhood, as well as to assess potential mediating effects.

## 2 Methods

### 2.1 Participants

The ABCD cohort is derived from an ongoing longitudinal multicenter study of the United States’ population. The data used in this study is part of release 2.0.1 of the ABCD cohort (Casey et al., 2018; Garavan et al., 2018). The ABCD cohort represents a cross-section of the American population of mixed race/ethnicity and diverse socioeconomic background. Almost 12,000 participants (47.8% female) between the ages of 8.9 and 11.1 years were included in the baseline assessment of the ABCD cohort (Supplementary Table S1); of those, we included up to 11,736 individuals in the brain-behavior analyses of the full cohort, and up to 4201 unrelated individuals of European ancestry in the PRS analyses, as described in section §2.4. The exact sample sizes for analyses involving neuroimaging vary mildly due to availability of structural T_1_-weighted and diffusion MRI scans, and exclusion of statistical outliers, as described in section §2.5.1.

A validation of the phenotypic findings was performed in the TwaalfMin (”12–Minus”) cohort. This is a cohort of childhood arrestees sampled from three different regions in The Netherlands; the cohort is enriched in individuals with externalizing behaviors that came into contact with the police before the age of 12 years (van Domburgh et al., 2009). Brain MRI scans and parent-reported CBCL were acquired for 150 participants (130 males and 20 females) at follow-up, when the participants were 18 years old on average (Supplementary Table S1); detailed information on the cohort and data acquisition is provided elsewhere (Cohn et al., 2012, 2016; Pape et al., 2015).

### 2.2 Externalizing behaviors

Behavior of the children was assessed with the parent-reported Child Behavior Checklist (CBCL) (Achenbach, 2001) for the broad externalizing behaviors scale (Ext-CBCL). The broad externalizing scale can be decomposed into domain-specific scales for aggressive (Agg-CBCL) and rule-breaking (Rb-CBCL) behavior (Supplementary Table S2). Each item on the CBCL was scored by the parents on a scale of 0–2 (0: never; 1: occasionally; 2: often; Supplementary Fig. S1). Raw summary scores for the scales were computed by summing the scores of the corresponding items on each scale. The score for the broad externalizing behaviors scale (Ext-CBCL) was defined as the sum of the two domain-specific scales (Agg-CBCL ​+ ​Rb-CBCL). Raw summary scores were used instead of sex- and age-normalized T-scores to allow for the investigation of sex- and age-related effects on the association of externalizing behaviors on brain structures and polygenic risk scores.

### 2.3 Magnetic resonance imaging (MRI)

#### 2.3.1 MRI acquisition parameters

MRI scans were acquired at the baseline assessment of the ABCD cohort. Full details on the acquisition parameters of the ABCD cohort are described elsewhere (Casey et al., 2018). In brief, high-resolution structural T_1_-weighted scans of the brain were acquired at 1.0 ​mm isotropic resolution. Multi-shell diffusion-weighted scans of the brain were acquired at 1.7 ​mm isotropic resolution in 96 diffusion directions with multiple b-values ranging from 500 to 3000. MRI scanning protocols were harmonized across acquisition sites and MRI scanners (Casey et al., 2018). Automated and manual quality control procedures were used to identify and exclude MRI scans of poor quality (Hagler et al., 2019).

For participants of the TwaalfMin cohort, structural T_1_-weighted and diffusion-weighted MRI scans were acquired on a Philips 3.0 ​T Intera MRI scanner. The structural T_1_-weighted anatomical scans consisted of 180 sagittal 1.0 ​mm slices, with an in-plane resolution of 1 ​× ​1 ​mm^2^ (FOV 256 ​× ​256 ​× ​180 ​mm^3^, TR 9.0 ms, TE 3.5 ms). The diffusion-weighted images (DWIs) were acquired using an 8-channel SENSE head-coil along 32 directions with a b-value of 1000 ​s/mm^2^ and four non-diffusion weighted (b ​= ​0) images; echo time (TE) 94 ms, repetition time (TR) 8135 ms, slice thickness 2.0 ​mm, slice gap 0.2 ​mm, field of view (FOV) 250 ​× ​250 ​× ​140.6 ​mm^3^, voxel size 2.0 ​× ​2.0 ​× ​2.2 ​mm^3^.

#### 2.3.2 Volumetric brain measurements

Volumetric brain measurements were obtained from FreeSurfer v5.3 reconstructions for the Desikan-Killiany cortical and aseg subcortical atlas (Fischl, 2012; Hagler et al., 2019). In this study, analyses were restricted to the cortical regions and subcortical structures previously used to investigate the association between gray matter volume and diagnosis of childhood disruptive behavior in the ABCD cohort (Waller et al., 2020) (Supplementary Table S4); we also included the rostral middle frontal gyrus previously reported to share a genetic factor with externalizing behavior during adolescent development (Teeuw et al., 2022). In addition, the following global brain measures were extracted: total brain volume (TBV), total cortical gray matter volume (CGM), total cortical gray matter surface area (CSA), mean cortical gray matter thickness (CTK), total subcortical gray matter volume (SCGM), and total cerebral white matter volume (CWM).

#### 2.3.3 White matter microstructural integrity brain measurements

White matter microstructural integrity measures were calculated for voxels of the brain using a diffusion tensor imaging (DTI) model (Basser et al., 1994; Pierpaoli and Basser, 1996), including fractional anisotropy (FA) and mean diffusivity (MD) (Alexander et al., 2007). The reconstruction for the ABCD cohort was based on a single shell (b ​= ​1000) of the diffusion-weighted scans to allow for better comparison to other developmental cohorts. The *AtlasTrack* software was used to automatically segment 37 major white matter tracts of the brain for participants in the ABCD cohort (Hagler et al., 2009, 2019). From those, we selected the 20 major white matter tracts also present in the John Hopkins University atlas; we also added the corpus callosum and fornix, for comparison to other developmental studies (Supplementary Table S5). The John Hopkins University atlas was used to extract white matter integrity measures for the 20 major white matter tracts of the brain of participants in the TwaalfMin cohort. The weighted average FA and MD were calculated for each white matter tract. In addition, the following global brain measures were extracted: total white matter tracts volume (wmVol), global fractional anisotropy (GFA), and global mean diffusivity (GMD) across all white matter tracts.

### 2.4 Genetic data and polygenic risk scores

Minimally processed subject-level genotype information of ABCD participants (N ​= ​10,659) were obtained from the ABCD Data Release 2.0.1 (https://doi.org/10.15154/1503209). Given the same-ethnicity requirement of PRS (i.e. between GWAS summary statistics and target cohort), the quality control and imputation procedures and further genetic analyses were restricted to participants of European descent.

Quality control was performed with the RICOPILI pipeline (Lam et al., 2020), where SNPs were removed if presenting minor allele frequency (MAF) ​< ​0.01, Hardy-Weinberg equilibrium values > 1E-6, and/or call-rate <0.98. We performed principal components analyses, selected only those who self-reported as ‘white’, and calculated the mean and standard deviation (SD) of this group. In order to exclude population outliers, we only kept in the study sample participants that did not exceed ±3 SD from the mean on either (or both) first 2 principal components (N ​= ​6564). Relatedness was estimated from the genetic data and only unrelated individuals were kept (pi-hat ≤0.20; N ​= ​5016). Individuals were kept if there was no evidence of sex-mismatch (i.e. between phenotypic and genetically determined), genotype missingness <5%, and autosomal heterozygosity deviation (FHET) within ±2 SD (N ​= ​4473). Following recommendations from the ABCD project, only individuals that were not part of genotype batch 461 and those that passed initial quality control were kept in the study sample (N ​= ​4397). Imputation was deployed from RICOPILI to the Michigan Imputation Server (https://imputationserver.sph.umich.edu), using the Haplotype Reference Consortium (HRC; hrc.r1.1.2016) data as reference. After imputation, RICOPILI recoded the imputed dosage data into best-guess genotypes, which were used for the PRS analyses if SNPs had imputation INFO score >0.8 and MAF >0.01.

Based on the output of recent GWAS for externalizing phenotypes, PRS were computed for each unrelated participant from European descent with available phenotypic data (i.e. Ext-CBCL and all covariates) in the ABCD cohort (N ​= ​4201). PRS were calculated as the sum of disease-/trait-associated alleles carried by an individual, weighted by the allele effect size in the discovery GWAS. Therefore, PRS can be interpreted as a measure of genetic risk due to common variants for the phenotype of the discovery GWAS. Here, we used four discovery GWAS: (i) the irritability GWAS from the UK Biobank cohort study (N_cases_ ​= ​104,545 and N_controls_ ​= ​264,687) (Watanabe et al., 2019), (ii) the broad spectrum of antisocial behaviors (ASB) GWAS from the Broad Antisocial Behavior Consortium (BroadABC) (N∼85,359) (Tielbeek et al., 2017, Tielbeek et al., 2021), (iii) the attention-deficit/hyperactivity disorder comorbid with disruptive behavior disorder (ADHD ​+ ​DBD) GWAS from the collaboration between the Danish iPSYCH cohort and the Psychiatric Genomics Consortium (PGC) (N_cases_ ​= ​3802 and N_controls_ ​= ​31,305) (Demontis et al., 2021), and (iv) the multivariate GWAS on traits related to self-regulation and addiction (N∼1.5 million) ([Bibr bib61][Bibr bib123]).

PRS were calculated using PRSice-2 (Choi and O’Reilly, 2019) software, using its default parameters for SNP clumping and filtering. PRS were computed based on different p-value thresholds (PRS_PT_) for each GWAS: 0.001, 0.05, 0.1, 0.2, 0.3, 0.4, 0.5, and 1.

### 2.5 Statistical analyses

#### 2.5.1 Data transformations, outliers, and covariates

A high proportion of participants scored low or zero on the summary scores of the CBCL, resulting in a skewed distribution of the data; a log-normal distribution described the data better than a normal distribution (Supplementary Fig. S2). The sum scores were therefore transformed with a 10-base logarithmic function (log_10_) prior to statistical analysis to reduce the skewness of the data. Note that for better interpretability, the regression coefficients reported in the main text have been transformed back to linear scale. Brain measures were normally distributed and Z-standardized prior to statistical analysis. Any extreme data points (i.e. outliers) more than 4 SD from the mean in the brain measures were automatically excluded from the analysis.

Analyses were corrected for sex and linear age effects. In addition, race/ethnicity (5 levels: “White”, “Black”, “Hispanic”, “Asian”, “Other”), parental education level (5 levels: “lower than highschool diploma”, “highschool diploma or general educational development”, “some college degree”, “bachelor degree”, “postgraduate degree”), household income (3 levels: “less than $50.000”, “between $50.000 and $100.000”, “more than $100.000”), parental marital status (2 levels: “married and/or living together”, “living apart”), and acquisition site (21 levels) were included as fixed effect covariates. Acquisition site was included as fixed effect covariate instead of random intercept effect to align the different statistical analyses across the capabilities of the software packages used in the analyses. Because diffusion MRI white matter integrity measures can be influenced by the amount of in-scanner head motion that is particularly prevalent in youth (Yendiki et al., 2014), mean motion was included as covariate for all analyses involving white matter integrity measures. In the genetic analyses that include the PRS, the self-reported race/ethnicity covariate was replaced by the first 10 ancestry-informed principal components that quantify the ethnic origins of the individual's ancestors.

#### 2.5.2 Phenotypic association between externalizing behavior and brain measures

We determined the phenotypic association between the Ext-CBCL score and the brain measures in the full sample of the ABCD cohort (Equation (1)). Linear mixed-effect regression models were used to control for the non-independence between related individuals. Results were corrected for multiple comparisons using the false discovery rate (FDR) method (Benjamini and Hochberg, 1995; Genovese et al., 2002); 24 tests for regional gray matter volume brain measures, 23 tests for regional fractional anisotropy brain measures, and 23 tests for regional mean diffusivity brain measures.

Equation 1 externalizing behavior_log_ ∼1 ​+ ​brain_Z_ ​+ ​sex ​+ ​age ​+ ​parental education ​+ ​household income ​+ ​marital status ​+ ​race/ethnicity ​+ ​ABCD acquisition site [ ​+ ​headmotion ]; with random intercept effect for family ID

#### 2.5.3 Associations with externalizing phenotype PRS

We tested the association of PRS derived from four distinct GWAS on externalizing phenotypes with the CBCL measures described above. These linear regression analyses were performed in PRSice2 software (Choi and O’Reilly, 2019), controlling for the effects of sex, age (in months), the first 10 ancestry informed principal components, and parental education level, household income, parental marital status, and acquisition site, as described above (Equation (2)). For each base-GWAS, the best-fit PRS for CBCL externalizing behavior (i.e. the most significantly associated PRS across the range of PRS_PT_ considered) was identified. In order to account for multiple testing and possible over-fitting effects, empirical p-values were obtained after 10,000 permutations. For visualization purposes only, participants of the ABCD cohort were then aggregated into five equally sized quantiles of increasing (best-fit) PRS and the phenotypic difference between the quantiles was depicted by plotting the behavior scores of each quantile compared to the median (reference) quantile. The computed PRS values (of the best-fit PRS_PT_) for each ABCD participant were taken forward for the analyses with brain measures.

Equation 2 externalizing behavior_log_ ∼1 ​+ ​PRS ​+ ​sex ​+ ​age ​+ ​parental education ​+ ​household income ​+ ​marital status ​+ ​first 10 ancestry informed principal components ​+ ​acquisition site

#### 2.5.4 Association of externalizing-related PRS with brain measures

We estimated the association between the three best-fitting PRS for externalizing phenotypes and the brain measures using linear regression models for the subset of unrelated participants of European descent that had imaging data and PRS available (Equation (3)). The same covariates as previously described were used, except that the self-reported race/ethnicity was replaced by the first 10 ancestry-informed principal components as determined by genetics.

Equation 3 Brain_Z_ ∼1 ​+ ​PRS_Z_ ​+ ​sex ​+ ​age ​+ ​parental education ​+ ​household income ​+ ​marital status ​+ ​first 10 ancestry informed principal components ​+ ​acquisition site [ ​+ ​headmotion ]

#### 2.5.5 Additive model for PRS and brain measures

Finally, the additive effect of both PRS and brain structures on externalizing behaviors was estimated to determine if the combined effect of both PRS and brain structures can explain more variance in externalizing behaviors than the two individually (Equation (4)). Multivariate models were examined to investigate the informative value for different sets of global and regional brain structures, with or without the four PRS.

Equation 4 externalizing behavior_log_ ∼1 ​+ ​PRS_Z_ ​+ ​brain_Z_ ​+ ​sex ​+ ​age ​+ ​parental education ​+ ​household income ​+ ​marital status ​+ ​first 10 ancestry informed principal components ​+ ​acquisition site [ ​+ ​headmotion ]

#### 2.5.6 Mediation analysis

A mediation analysis was performed to determine if the association between PRS and externalizing behaviors was mediated through alterations in brain structures. The mediation model was only applied in a post-hoc fashion only to associations for which mediation was deemed plausible; i.e. there was a significant total effect (i.e., a significant association of a PRS with the externalizing behavior score) and a significant association between the PRS and a brain structure. The mediation analysis was performed using the PROCESS macro version 4.0 (Andrew Hayes, New York: The Guilford Press 2018) in SPSS version 23 (IBM Corp. Released 2015. IBM SPSS Statistics for Windows, Version 23.0. Armonk, NY: IBM Corp.). The same covariates as previously described were regressed from the variables prior to inputting them into mediation modeling. A parallel mediation model was tested in cases with multiple potential mediators for the same PRS. The significance of the effect sizes was determined by bootstrapping.

#### 2.5.7 Statistical testing

Significance of the predictors and covariates of interest in the regression models was determined by comparing the models to a null model where the regression coefficient of that particular predictor or covariate was constrained to zero. Results for phenotypic association between Ext-CBCL score and the nine global brain measures were corrected for multiple comparisons using Bonferroni correction at significance threshold *p*_bonferroni_ ​= ​0.05/9 ​= ​0.0056. Results for the phenotypic association between Ext-CBCL score and the local brain measures were corrected for multiple comparisons using the FDR method at *q* ​= ​0.05 (Benjamini and Hochberg, 1995; Genovese et al., 2002); 24 tests for regional gray matter volume brain measures, 23 tests for regional fractional anisotropy brain measures, and 23 tests for regional mean diffusivity brain measures. The same approach was used to correct for multiple testing in the associations between PRS and regional brain measures.

#### 2.5.8 Post-hoc analyses

A sensitivity analysis was performed for the effect of the global brain measures on the associations between externalizing behaviors and the regional brain measures by including the relevant global brain measure (i.e. total brain volume for gray matter volumes, and global FA or global MD for white matter integrity) in the regression models as fixed effect on the means. In addition, we performed a sensitivity analysis of the associations between externalizing behaviors and the brain measures for the subset of participants of European descent used in the PRS analyses. For significant associations between Ext-CBCL score and brain measures or PRS, a post-hoc analysis was performed to determine if the association was primarily due to any of the domain-specific subscales (Agg-CBCL and Rb-CBCL).

#### 2.5.9 Validation in TwaalfMin childhood arrestees cohort

We repeated the phenotypic analyses in the independent TwaalfMin cohort to determine whether the associations between externalizing behaviors and brain measures found in the ABCD cohort during late childhood were also present during late adolescence/early adulthood in a group of participants enriched in externalizing behaviors (mean CBCL externalizing behavior score ​= ​9.5; Supplementary Table S1).

## 3 Results

### 3.1 Externalizing behaviors in late childhood

The endorsement of externalizing behaviors in children by their parents was low overall in the ABCD cohort (Supplementary Table S3; Supplementary Fig. S1; Supplementary Fig. S2; **Supplementary Data File F1**), as would be expected for a cohort that represents a cross-section of a population of typically developing children. There was a small but significantly higher externalizing score reported for boys than for girls (up to +0.25 points; *p*_unc_ ​= ​9.29E–34; Supplementary Table S3).

There was a significant negative effect for household income (up to −0.18 points), parental education level (up to −0.16 points), and marital status (up to −0.12 points) on the externalizing score (*p*_unc_ < 3.18E–03; Supplementary Table S3); i.e. there were slightly less externalizing behaviors on average slightly lower in families with a higher household income, a higher education level of the parents, and parents who were married and/or living together. There was a significant overall effect of race/ethnicity (between −0.06 and −0.28 points) on externalizing score (*p*_unc_ < 2.53E–07; Supplementary Table S3); i.e. children of white ethnicity exhibited higher externalizing behaviors than children from black, hispanic, or asian ethnicity. Of note, there was a significant overall effect of the acquisition site on the externalizing score that needed to be accounted for in the analysis (*p*_unc_ < 5.43E–14), but this may also represent unknown geographic-dependent environmental effects (e.g. level of urbanicity).

Together, the seven covariates explained up to 7.7% of the variance in externalizing behaviors (Supplementary Table S3). The strongest predictors of externalizing behaviors were parental education and household income (approximately 2.3% combined due to collinearity), followed by sex (1.5%) and acquisition site (1.5%). Age, marital status, and race/ethnicity contributed least (<0.5%).

Post-hoc analysis of the aggressive and rule-breaking scores on the subscales of externalizing behaviors revealed that externalizing behaviors in the ABCD cohort in 9–10 year olds were primarily driven by aggressive-related behavior, although both traits showed a highly similar pattern of effects from covariates (Supplementary Table S3). In addition, there was a strong phenotypic correlation between the two subscales (*r*_ph_ ​= ​+0.65 [+0.61; +0.68]; *p* ​= ​1.18E–131).

### 3.2 Phenotypic associations between externalizing score and brain structures

#### 3.2.1 Global brain structures

The volumes of global brain structures (total, cortical gray, subcortical gray, and cerebral white matter) were associated with a reduction of the Ext-CBCL scores (*p*_unc_ < 3.28E–07; Table 1; **Supplementary Data File F1**). Mean cortical gray matter thickness or global fractional anisotropy and mean diffusivity of white matter tracts were not significantly associated with Ext-CBCL scores. In general, global brain structures explained up to 0.56% of additional variance of externalizing behaviors on top of the demographic covariates. All results except for total subcortical gray matter volume remained significant in the subsample used in the PRS analyses. The post-hoc analyses of the scores on the Agg-CBLC and Rb-CBCL subscales revealed that the effects for Ext-CBCL were about equally driven by Agg-CBCL and Rb-CBCL.

**Table 1:** Phenotypic associations between the score for the externalizing behaviors scale of the Child Behavior Checklist (CBCL) and global brain structures.

Brain structure	Regression coefficient a	Variance explained b	Significance p-value c	Sensitivity analysis: subsample d	Post-hoc analysis: Aggression or rule-breaking e
Total brain volume	−0.075	0.63%	**1.16E–11**	✔	Agg & Rb
Total cortical gray matter volume	−0.077	0.69%	**5.04E–13**	✔	Agg & Rb
Total cortical gray matter surface area	−0.086	0.85%	**6.60E–15**	✔	Agg & Rb
Mean cortical gray matter thickness	−0.002	0.00%	7.54E–01 [n.s.]	n.a.	n.a.
Total subcortical gray matter volume	−0.054	0.32%	**3.28E–07**	✔	Agg & Rb
Total cerebral white matter volume	−0.052	0.30%	**9.63E–07**	✔	Agg & Rb
Total white matter tracts volume	−0.050	0.21%	**3.18E–06**	✖	Agg & Rb
Global fractional anisotropy	−0.017	0.02%	1.82E–01 [n.s.]	n.a.	n.a.
Global mean diffusivity	−0.000	0.00%	9.84E–01 [n.s.]	n.a.	n.a.

a Partially standardized effect size reporting the change in score on the behavioral scale (points) for one standard deviation change in brain measure after conversion back to linear scale.

b Percentage of total phenotypic variance explained in the score on the externalizing behaviors scale.

c Uncorrected p-value (p_unc_) that remained significant after correction for multiple comparisons (9 tests for global brain structures) with the Bonferroni method at p_bonferroni_ < 0.05/9 ​< ​0.0056.

d *Effects that remained nominally significant (p*_*unc*_*<0.05) in the subsample of participants with polygenic risk scores available are marked with a check mark (*✔)*.*

e Post-hoc analyses to determine if the significant association was primarily due to the score on the CBCL aggressive behavior subscale (Agg-CBCL) or due to the score on the CBCL rule-breaking behavior subscale (Rb-CBCL).

#### 3.2.2 Regional (sub)cortical gray matter volumes

All 24 lateralized (sub)cortical gray matter structures were significantly associated with the Ext-CBCL score at *p*_fdr_ < 0.05, including their association with the Agg-CBCL and Rb-CBCL subscales (**Supplementary Data File F1**). Five cortical structures remained significant after correcting for the effect of total brain volume, indicating that these regions contributed to externalizing behavior scores above and beyond the effect of total brain volume (Table 2). Specifically, the lower gray matter volume of the right caudal anterior cingulate cortex, the left rostral middle frontal gyrus, the left superior frontal gyrus, the left superior temporal gyrus, and the left insular cortex were significantly associated with the Ext-CBCL score (B ​= ​−0.045 to −0.065; *p*_unc_ < 6.67E–07; *p*_fdr_ < 1.46E–06; Table 2; **Supplementary Data File F1**; after correction for the effect of total brain volume B ​= ​−0.038 to −0.016; *p*_unc_ < 4.70E–02; **Supplementary Data File F1**). All five cortical regions replicated in the subsample used in the PRS analyses, and all were associated with the Agg-CBCL and Rb-CBCL subscales in the post-hoc analysis. However, after controlling for total brain volume, the cingulate and frontal regions were only associated with the Agg-CBCL but not the Rb-CBCL subscale, and the temporal region and insular cortex were only associated with the Rb-CBCL but not Agg-CBCL subscale (**Supplementary Data File F1**).

**Table 2:** Phenotypic associations between the score on the externalizing behaviors scale of the Child-Behavior Checklist (CBCL) and regional brain structures that remained significant after controlling for global brain measures.

Brain structure	Regression coefficient a	Variance explained b	Significance p-value c	Sensitivity analysis: correction for global brain measures d	Sensitivity analysis: subsample e	Post-hoc analysis: Aggression or rule-breaking f
Right caudal anterior cingulate gyrus	−0.045	0.22%	**6.67E–07**	✔	✔	Agg & Rb
Left rostral middle frontal gyrus	−0.060	0.34%	**9.27E–10**	✔	✔	Agg & Rb
Left superior frontal gyrus	−0.065	0.40%	**3.26E–11**	✔	✔	Agg & Rb
Left superior temporal gyrus	−0.058	0.31%	**4.12E–09**	✔	✔	Agg & Rb
Left insular cortex	−0.060	0.34%	**6.84E–10**	✔	✔	Agg & Rb

*Note that associations of all regional (sub)cortical gray matter brain structures and all volumes of white matter tracts were significant at p*_*fdr*_*< 0.05 prior to controlling for global brain measures (see****Supplementary Data File F1****). This table only shows (sub)cortical gray matter brain structures that remained significant at uncorrected p*_*unc*_*< 0.05 after controlling for the global brain measures.*

a Partially standardized effect size reporting the change in score on the behavioral scale (points) for one standard deviation change in brain measure after conversion back to linear scale.

b Percentage of total phenotypic variance explained in the score on the externalizing behavior scale.

c Uncorrected p-value (p_unc_) that remained significant after correction for multiple comparisons with the FDR method and remained nominally significant after controlling for the effect of global brain.

d *Effects that remained nominally significant (p*_*unc*_*<0.05) after controlling for the effect of the global brain are marked with a check mark (*✔)*.*

e *Effects that remained nominally significant (p*_*unc*_*<0.05) in the subsample of participants with polygenic risk scores available are marked with a check mark (*✔)*.*

f Post-hoc analysis to determine if the significant association was primarily due to the score on the CBCL aggressive behavior subscale (Agg-CBCL) or due to the score on the CBCL rule-breaking behavior subscale (Rb-CBCL).

#### 3.2.3 Regional microstructural integrity and volume of white matter tracts

Fractional anisotropy (FA) of the corpus callosum, left corticospinal tract, left fronto-occipital fasciculus, and left uncinate fasciculus were significantly associated with the Ext-CBCL score at uncorrected *p*_unc_ < 0.05, while none of the mean diffusivity (MD) of the regional white matter tracts were (**Supplementary Data File F1**). The significant effects were primarily due to aggressive behavior (**Supplementary Data File F1**). However, none survived correction for multiple comparisons at *p*_fdr_ < 0.05. In contrast, the volumes of the white matter tracts (wmVol) were all significantly associated with reductions in the scores on the Ext-CBCL scale (as well as the scores on the Agg-CBCL and Rb-CBCL subscales), and all survived correction for multiple comparisons (all *p*_fdr_ < 0.029), but these effects could all largely be explained by total brain volume and were no longer significant after correcting for total brain volume (all *p*_unc_ > 0.05; **Supplementary Data File F1**).

### 3.3 Validation of phenotypic associations in the TwaalfMin cohort

We attempted to validate our findings of significant phenotypic associations between Ext-CBCL score and brain morphology in the ABCD cohort (see results in Table 1; Table 2) in an independent sample from the TwaalfMin cohort – a cohort of childhood arrestees enriched for individuals with externalizing behaviors with neuroimaging assessment at age 18 years. The correlation between the effect sizes were reasonably consistent between the two cohorts for global brain measures (pearson's ***ρ*** ​= ​+0.72) and white matter fractional anisotropy (***ρ*** ​= ​+0.38), but less so for gray matter volume (***ρ*** ​= ​+0.16) or mean diffusivity (***ρ*** ​= ​+0.00). Most of the findings in the TwaalfMin cohort did not reach significance (**Supplementary Data File F1**).

### 3.4 Associations between externalizing behaviors and polygenic risk scores for externalizing phenotypes

**Fig. 1:**
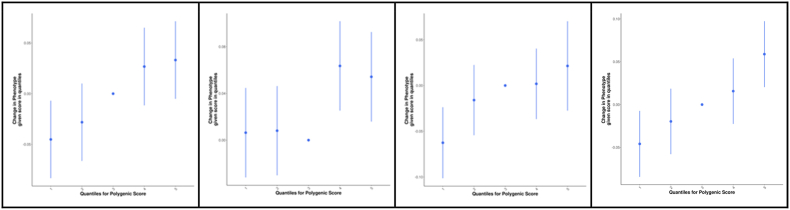
Association of polygenic risk scores (PRS) based on the (A) attention-deficit/hyperactivity disorder comorbid with disruptive behavior disorders (ADHD ​+ ​DBD), (B) antisocial behavior (ASB), (C) irritability, and (D) traits related to self-regulation & addiction GWAS with scores on the externalizing behavior scale of the Child Behavior Checklist (Ext-CBCL) in 4201 children from the Adolescent Brain and Cognitive Development (ABCD) cohort. The bar plots (top-row) display the linear regression results of the PRS computed across a range of P-value thresholds (x-axis), with the variance explained (R^2^) indicated on the y-axis and uncorrected P-values displayed on top of each bar. The best-fit P-value threshold (and corresponding number of SNPs included in that threshold and empirical P-value) were 0.1 (35664 SNPs; empirical P ​= ​0.0002), 1 (207771 SNPs; empirical P ​= ​0.003), 0.3 (133697 SNPs; empirical P ​= ​9.999E-05), and 0.1 (47059 SNPs; empirical P ​= ​9.999E-05) for ADHD ​+ ​DBD-PRS, ASB-PRS, irritability-PRS, and traits related to self-regulation and addiction-PRS, respectively. Quantile plots (bottom-row) show how the Ext-CBCL scores vary with increasing PRS (using the identified best-fit PRS). The x-axis shows five equally sized quantiles, and the y-axis shows the difference in the externalizing behavior scores (in terms of regression coefficient and standard error bars) when comparing with the median (reference) quantile.

Scores on the Ext-CBCL scale, and its subscales Agg-CBCL and Rb-CBCL, were significantly associated with the PRS based on all four GWAS for the externalizing phenotypes, namely comorbid ADHD ​+ ​DBD, ASB, irritability, and traits related to self-regulation & addiction (Fig. 1; Supplementary Table S6).

### 3.5 Associations between externalizing PRS and brain structures

Among the global brain structures, total cortical gray matter volume was significantly associated with the PRS based on the comorbid ADHD ​+ ​DBD GWAS (*B ​=* −0.067; *p*_unc_ ​= ​2.72E–02; **Supplementary Data File F1**), global FA was associated with the PRS based on the irritability GWAS (*B ​=* −0.105; *p*_unc_ ​= ​1.31E–02; **Supplementary Data File F1**), and total subcortical gray matter volume, total cerebral white matter volume and total volume of white matter tracts was associated with the PRS based on addiction and self-regulation (*B* ​= ​+0.067; *p*_unc_ < 4.46E–02; **Supplementary Data File F1**). In addition, FA of the left and right cingulum bundles was significantly associated with the PRS based on the irritability GWAS (*B* ​= ​−0.139 to −0.145; *p*_unc_ < 5.41E–03; *p*_fdr_ < 4.15E–02; **Supplementary Data File F1**), and the volume of the left inferior longitudinal fasciculus was significantly associated with the PRS based on addiction and self-regulation (*B* ​= ​+0.114; *p*_unc_ ​= ​1.45E–03; *p*_fdr_ ​= ​3.33E–02; **Supplementary Data File F1**).

### 3.6 Additive effect of externalizing PRS and brain structures

PRS and brain structures acted for the most part independently in explaining variance in the Ext-CBCL score and the Agg-CBCL and Rb-CBCL subscales (**Supplementary Data File F1**). The null model (i.e. only sociodemographic factors and covariates; Equation (1)) explained ∼7% of the variance of the Ext-CBCL in the subsample of participants from European descent with imaging data and PRS available (Supplementary Table S7); note that this was slightly lower than in the full ABCD sample described above. The models that included one of the brain structure (Equation (2)) explained additional variance ΔR^2^ up to +0.67% (and ΔR^2^ up to +0.26% after controlling for global brain measures), and the models that included one of the PRS (Equation (3)) explained additional variance ΔR^2^ up to +0.50% of the variance. When combined into an additive model (Equation (4)), the brain measure and PRS together explained up to +1.09% additional variance ΔR^2^. A multivariate model with all brain measures and PRSs together explained up to +4.95% additional variance ΔR^2^ (Supplementary Table S7).

### 3.7 Mediation of the association between polygenic risk scores and externalizing behaviors through brain structures

**Fig. 2:**
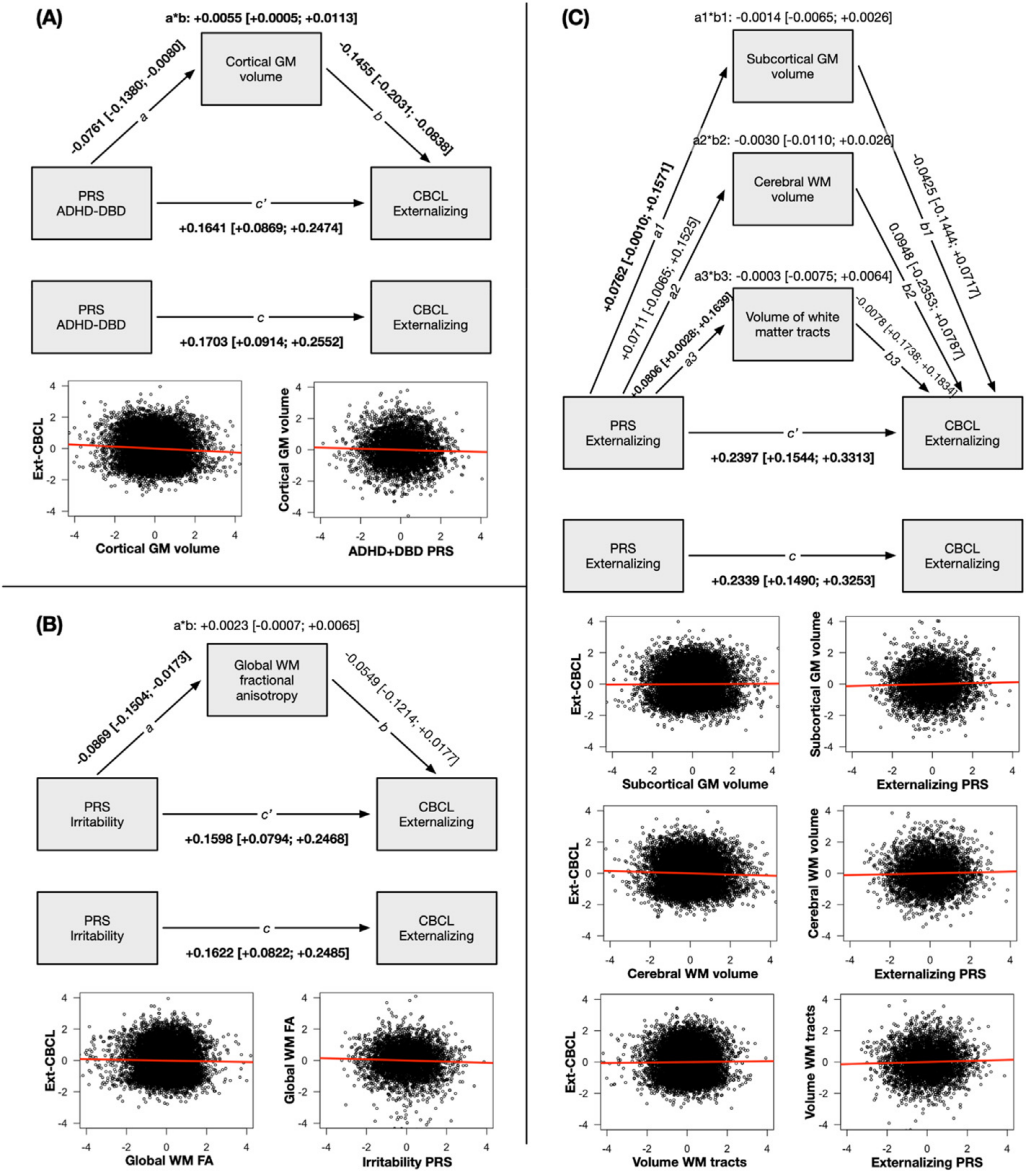
Mediation of the association between polygenic risk scores (PRS) for (**A**) ADHD ​+ ​DBD, (**B**) Irritability, and (**C**) Externalizing with parent-reported externalizing behaviors on the Child Behavior Checklist (CBCL) through global brain structures. Significant effects at uncorrected *p*_unc_ < 0.05 are printed in **boldface**. The total effect (*c*) of PRS on externalizing behaviors was decomposed into a direct effect (*c*’) and an indirect effect (*a*∗*b*), where the indirect path was mediated through a brain structure. Reported values on the paths of the mediation analysis are the effect size and bootstrapped 95% confidence intervals.

The significant associations between three PRS and five global brain structures were eligible for further analysis in a mediation model. Total cortical gray matter volume mediated the association between ADHD ​+ ​DBD PRS and Ext-CBCL (indirect effect: +0.0055, *p*_*unc*_ ​= ​4.89E–02; Fig. 2; **Supplementary Data File F2**). Global white matter fractional anisotropy did not significantly mediate the association between irritability PRS and Ext-CBCL (indirect effect: +0.0023, *p*_*unc*_ ​= ​0.225 [n.s.]; Fig. 2; **Supplementary Data File F2**). Neither did subcortical gray matter volume, cerebral white matter volume, or volume of the white matter tracts mediate the association between externalizing PRS and Ext-CBCL (indirect effect: range −0.0003 to −0.0030; *p*_unc_ > 0.389 [n.s.]; Fig. 2; **Supplementary Data File F2**).

## 4 Discussion

We investigated to which extent genetic and brain structural variation were associated with externalizing behaviors in the large ABCD study cohort of typically developing children aged 9–10 years old. The main findings were that increasing levels of externalizing behaviors were associated with lower global and regional gray matter brain volumes. The significant findings for gray matter measures observed in the ABCD cohort could not be validated in the much smaller independent TwaalfMin validation cohort of late adolescent individuals enriched in externalizing behaviors, except for a good correlation among some of the effect sizes between the two cohorts. The significant associations in ABCD were largely explained by effects of total brain volume. Externalizing behavior was not significantly associated with global white matter integrity after correction for multiple testing in either cohort. The PRS based on different externalizing phenotypes (i.e. ASB, irritability, comorbid ADHD ​+ ​DBD, and addiction & self-regulation) were significantly associated with externalizing behaviors and with global brain volumes in children from the ABCD cohort. When combining brain measures and PRS into a single model, our results suggested that genetics and brain structural variation had unique contributions to externalizing behaviors. However, we did find that total cortical gray matter volume partially mediated the association between the ADHD ​+ ​DBD PRS and externalizing behaviors in late childhood.

We found that lower global brain volumes, cortical surface area, and thickness were associated with higher levels of externalizing behaviors in children of the ABCD study cohort. Previous studies have reported lower intracranial and global gray and white matter volumes in children and adolescents with ADHD compared to controls (Boedhoe et al., 2020; Hoogman et al., 2017; van Ewijk et al., 2012), and lower total brain volume in children with callous unemotional traits (Bolhuis et al., 2019b). The association found between cortical surface area and externalizing behaviors has been previously reported in the ABCD cohort (Modabbernia et al., 2022) and in other cohorts, including decreased cortical thickness (Carlisi et al., 2020; Romer et al., 2021). The regional gray matter volumes found associated with externalizing behavior in late childhood have previously been implicated in externalizing behaviors in youth and adults, including for the ABCD cohort (Cohn et al., 2016; Durham et al., 2021; Raine et al., 2000; Teeuw et al., 2022; Waller et al., 2020). The limited validation in the TwaalfMin cohort suggests that although there is a certain level of agreement on the direction and magnitude of the effect sizes between the two cohorts that span two different stages of childhood and adolescent development, the effect sizes are generally small for broadscale externalizing behaviors.

Most of the associations of externalizing behaviors with regional gray matter volume could be explained by total brain volume, as has been reported before (Durham et al., 2021). In contrast, our results on the association of white matter integrity and externalizing behaviors did not survive correction for multiple testing (similarly to (Cardenas-Iniguez et al., 2020) in the same cohort), and, in that sense, do not replicate previous significant associations reported by others and our own work (Bolhuis et al., 2019a, 2019b; Muetzel et al., 2018; Pape et al., 2015; Raine et al., 2003; Teeuw et al., 2022; Waller et al., 2017). In our previous findings, in an independent longitudinal typically developing twin cohort, externalizing behavior was most prominently associated with global white matter integrity around mid-adolescence, and less so in early or late adolescence (Teeuw et al., 2022). Therefore, it is possible that an age-dependent or developmental-stage-dependent association between brain white matter integrity and externalizing behavior is present. We have previously witnessed a similar effect of an age-dependent association between cognition and cortical thickness, where the association was no longer significant around the end of adolescence and beginning of adulthood when the direction of the association reversed (Schnack et al., 2015). More evidence for a potential cross-over effect should come from longitudinal studies (Crone and Elzinga, 2015).

Our results with PRS based on GWAS for all three externalizing-related phenotypes, which includes one derived exclusively from adult individuals (i.e. PRS-irritability), were significantly associated with externalizing behaviors in children from the ABCD study cohort. Although the phenotypic variance explained by the PRS was quite small, this is typical for PRS results. In a previous study, a PRS based on childhood aggression explained 0.44% of the phenotypic variance in aggression in a sample of 7-year-old Dutch children, for example (Ip et al., 2021). The PRS on broadscale ASB was found to explain up to 3.9% of the liability to externalizing psychopathology at age 18 years (Tielbeek et al., 2021). Other studies have also reported on the relationship between PRS for aggressive behavior or ADHD with childhood aggression or externalizing behaviors (Kretschmer et al., 2021; Luo et al., 2022; Wainberg et al., 2022). Even so, our PRS results distinguished the fraction of individuals with higher genetic risk for externalizing-related phenotypes, which demonstrated higher levels of externalizing behaviors, from those with lower genetic risk load (who display lower levels of externalizing behavior; as shown in Fig. 1). Interestingly, the PRS based on adults was found to be associated with externalizing behavior in these children, suggesting that the genetic risk is at least in part stable with age. This notion is in line with a previous study that reported that the genetic factors on aggressive behavior expressed in childhood continue to play a role later in life (van der Laan et al., 2021). All four PRS predicted parent-reported externalizing behavior in children, although the PRS were based on different externalizing-related phenotypes, including clinically defined ADHD comorbid with DBD, broad measures of ASB in people with and without psychiatric diagnoses, as well as irritability and addiction & self-regulation. These results can indicate shared genetic mechanisms between the different types of externalizing behaviors, similar to what has been reported for psychiatric disorders (Akingbuwa et al., 2022; Jones et al., 2018).

We also tested the associations of PRS for externalizing phenotypes with brain structural variation. Five significant associations were found between PRS and global brain structures: for ADHD ​+ ​DBD PRS with total cortical gray matter volume, for irritability PRS with global white matter fractional anisotropy, and for addiction & self-regulation PRS with total subcortical gray matter volume, total cerebral white matter volume and total volume of white matter tracts. A fraction of the total effect of the association between ADHD ​+ ​DBD PRS with externalizing behavior could be attributed to an indirect effect mediated through cortical gray matter volume. To the best of our knowledge, our study is the first to measure associations of PRS for externalizing behaviors with gray and white matter structures throughout the brain. So far, there is one previous study on the PRS for ASB, based on an earlier version of the ASB GWAS from the BroadABC (Tielbeek et al., 2017), focussing on the 3-dimensional shape of the amygdala only, reporting an association in a cohort of ADHD patients (Kleine Deters et al., 2022). Our findings confirm and extend findings from twin and family studies and a GWAS study. It has been known for quite some time that adolescent externalizing behaviors and brain structural variation are in part heritable (Blokland et al., 2012; van der Laan et al., 2021). A few studies have reported a partly shared genetic etiology between externalizing behaviors and brain structural variations in adolescent twins (Coccaro et al., 2018; Rijsdijk et al., 2010; Teeuw et al., 2022). An overlap in genetic loci between a GWAS for aggression (EAGLE consortium) (Pappa et al., 2016) and a GWAS for subcortical brain volumes (Satizabal et al., 2019) found two genes associated with the volumes of the nucleus accumbens and the amygdala (van Donkelaar et al., 2018). Moreover, common genetic variants for the genetic risk of phenotypes which show some overlap with externalizing behavior but were not used in our current study (e.g., ADHD) have been found related to smaller total brain volume and intracranial volume (Klein et al., 2019; Mooney et al., 2020). Together with our results, this suggests that at least part of genetic variants responsible for externalizing phenotypes also influence brain structural variation, of which part of the genetic influence on externalizing phenotypes is mediated through total cortical gray matter volume.

In the current study, brain structures and PRS each contributed up to 0.5% of the variance of externalizing behaviors, for a combined total of up to approximately 1.0%, or up to 5% when combined in a multivariate model. Please note that the influence of child's age (explaining <0.1% of individual variation in externalizing behavior), child's sex (∼1.4%), family household situation (∼1.9%), ethnic background (∼0.3%), and acquisition site (∼1.6%) was already accounted and thus controlled for in these analyses. Together, all these sociodemographic factors explained about 7% of the individual variation in externalizing behaviors. Thus, while the contributions of neuroimaging and genetics appear to be small, they add significantly to the proportion of explained variance in externalizing behaviors. Moreover, our results support the idea that the significant influence of common genetic variation on externalizing behaviors is mediated through brain structures in children around age 10 years.

About 90% of the individual variation in externalizing behaviors is still left unaccounted for, implying unknown factors of genetic and/or environmental origin. One possibility is that the genetic effects on externalizing behaviors are mediated through brain function instead of brain structure, as has been suggested for adults (Sadeh et al., 2019). A previous study has reported on the link between functional connectivity in major brain networks and externalizing behaviors for the ABCD cohort (Wainberg et al., 2022). Alternatively, abnormal brain structural variation might be more prominent when moving toward more extreme forms of externalizing behaviors, such as psychopathy, which has previously been associated with brain structures in the TwaalfMin cohort (Cohn et al., 2016; Pape et al., 2015). Another possibility is that the environment, or exposome, plays an important role; e.g., environmental factors may interact with gene expression in externalizing behaviors (Lin et al., 2022; Pries et al., 2022; Vermeulen et al., 2020; Weeland et al., 2015), possibly through epigenetic mechanisms (Palumbo et al., 2018; van Dongen et al., 2021). For example, the acquisition site in this multicenter study explains more variance than neuroimaging and genetics combined. The geographic location of the acquisition site could act as a proxy to variation in demographics, or represent environmental factors (e.g. level of urbanicity, neighborhood poverty, or exposure to toxins such as lead or air pollutants) that affect the families living in the surrounding area (Legrand et al., 2008; Maxwell et al., 2022; Peterson et al., 2015). Family and social environmental factors can also play an important role in the development and prevention of externalizing behaviors (Gong et al., 2021; Kretschmer et al., 2022; Pandey et al., 2018; Sijtsema and Lindenberg, 2018). Studying environmental factors and their interaction with genetics and neuroimaging could aid in identifying missing pieces of the pathways to externalizing behaviors.

## 5 Limitations

There are several limitations to this study that have to be taken into consideration when interpreting these findings. First, this analysis was performed in a large cohort of typically developing children (N∼11,878), but only within a very small time window of childhood development (age range 9–10 years old). The independent validation cohort might have been underpowered (N∼150). Second, the measures of externalizing behaviors were based on parent reports only. While parent reports are generally considered to have good reliability, self- and teacher-reported behavior may offer a unique perspective of a child's behavior unbeknownst to the parents. Third, we limited the analysis of gray matter brain volumes to those regions previously implicated in association with externalizing behaviors. As such, we do not know if other gray matter brain structures might be associated with externalizing behavior in this cohort. Fourth, the analysis of the PRS was based on a subset of participants of European descent (N∼4201). The results of the associations with PRS may therefore not generalize to populations of other ancestries. Despite this reduction in sample size for the genetic analyses, most results for the phenotypic association between externalizing behavior and brain structures were replicated in the sensitivity analysis. In addition, a post-hoc power analysis indicated that effect sizes (in terms of correlation) as low as ±0.047 could be detected with 90% certainty at an uncorrected significance threshold of *p* ​= ​0.05 with this sample size. Additionally, different PRS methods exist and more recent methods, that more formally model the genetic architecture, tend to perform better than traditional p value–based clumping and thresholding methods, such as PRSice-2 (Ni et al., 2021). Alternative PRS approaches would potentially also allow for inclusion of related individuals in the PRS analyses to improve the power of the study. However, not using the latest method does not necessarily invalidate our results (and those in previous publications), since we do use a well-established and still widely used method.

## 6 Conclusion

The association between externalizing behaviors and gray matter brain structures appears to be mostly driven by the global effect of the total brain volume. Our results from the additive modeling suggest that both PRS for externalizing phenotypes and gray- and white matter brain structures contribute to externalizing behaviors in late childhood, with some evidence for mediation of PRS through brain structures. However, as a large proportion of individual variation in externalizing behavior remains unidentified, brain function and interaction effects with the environment are believed to be another potent source of additional variation. The study has shown that the combined effort of research from genetics and neuroimaging can be informative in understanding the onset of externalizing behaviors in late childhood.

## Funding

This research was supported by funding from the Dutch Research Agenda (NWA) NeurolabNL project (grant number 400-17-602), and supported by the 10.13039/501100002658Consortium on Individual Development (CID) that is funded through the Gravitation program of the Dutch Ministry of Education, Culture, and Science and the Netherlands Organization for Scientific Research (10.13039/501100003246NWO grant number 024.001.003). MK was supported by a Rubicon grant (no. 45219212) from the Dutch Research Council.

## Declaration of competing interest

The authors declare that they have no known competing financial interests or personal relationships that could have appeared to influence the work reported in this paper.
