# Supplementary material for: Polygenic risk scores and brain structures both contribute to externalizing behavior in childhood - A study in the Adolescent Brain and Cognitive Development (ABCD) cohort
Source: Neurosci Appl. 2023 May 24;2:101128. doi: 10.1016/j.nsa.2023.101128 (PMC12244044; doi:10.1016/j.nsa.2023.101128)
Supplement: Multimedia component 3 [file mmc3.docx]

# Supplementary Information

## Cohort descriptions

The main analysis was performed in the ABCD cohort, a cross-section of the American population of mixed race/ethnicity and diverse socioeconomic background, with a validation of the phenotyping findings in the TwaalfMin (“12-Minus”) cohort, a cohort of childhood arrestees enriched with externalizing behaviors from three regions in The Netherlands, that came into contact with the police before the age of 12 years (**Supplementary Table S1**).

**Supplementary Table S1.** Demographics of the Adolescent Brain and Cognitive Development (ABCD) and TwaalfMin cohort.

| **Measure** | **ABCD cohort** | **TwaalfMin cohort** |
| --- | --- | --- |
| **Participants** (count) | 11,878 | 150 |
| **Age** (years) ^a^ | 9.9±0.6  [8.9; 11.1] | 17.6±1.6  [12.0; 20.5] |
| **Sex** (female:male) | 5,682:6,196  47.8% female | 20:130  13.3% female |
| **Race/ethnicity** | 52.1% white  15.0% black  20.3% Hispanic  2.1% Asian  10.5% other | 66.7% Dutch  33.3% non-Dutch |
| **Household income** | 29.7% less than $50.000  28.3% between $50.000 and $100.000  42.0% more than $100.000 | 37.3% low income  41.3% middle income  20.0% high income |
| **Parental education level** | 5.0% lower than high school diploma  9.5% high school diploma or general educational development  26.0% some college degree  25.4% bachelor degree  34.1% postgraduate degree | N/A |
| **Parental marital status** | 73.7% married and/or living together  26.3% living apart | N/A |
| **CBCL externalizing** ^a^ | 4.45±5.9 [0; 49] | 9.53±9.7 [0; 41] |
| **CBCL aggressive** ^a^ | 3.26±4.4 [0; 36] | 4.81±5.5 [0; 22] |
| **CBCL rule-breaking** ^a^ | 1.19±1.9 [0; 20] | 4.71±4.7 [0; 21] |

^a^ The mean±SD and range [minimum; maximum] are reported for age in years and the untransformed raw scores on the parent-reported CBCL.
Abbreviations (in alphabetical order): CBCL = Child Behavior Checklist; s.d. = standard deviation from the mean.

## Externalizing behavior on the Child Behavior Checklist

Externalizing behavior of the children was assessed by the parent-reported Child Behavior Checklist (CBCL) [(Achenbach, 2001)](https://paperpile.com/c/enbthS/xGlp). The broad externalizing behavior scale (Ext) can be decomposed into aggressive behavior (Agg) and rule-breaking behavior (Rb) subscales (**Supplementary Table S2**).

**Supplementary Table S2**. Items on the broad externalizing behavior scale of the Child Behavior Checklist.

| **Aggressive behavior subscale** | | **Rule-breaking behavior subscale** | |
| --- | --- | --- | --- |
| **Item** | **Description** | **Item** | **Description** |
| 3 | Argues a lot | 2 | Drinks alcohol without parents’ approval |
| 16 | Cruelty, bullying, or meanness to others | 26 | Doesn’t seem to feel guilty after misbehaving |
| 19 | Demands a lot of attention | 28 | Breaks rules at home, school, or elsewhere |
| 20 | Destroys his/her own things | 39 | Hangs around with other who get in trouble |
| 21 | Destroys things belonging to his/her family or others | 43 | Lying or cheating |
| 22 | Disobedient at home | 63 | Prefers being with older kids |
| 23 | Disobedient at school | 67 | Runs away from home |
| 37 | Gets in many fights | 72 | Sets fires |
| 57 | Physically attacks people | 73 | Sexual problems |
| 68 | Screams a lot | 81 | Steals at home |
| 86 | Stubborn, sullen, or irritable | 82 | Steals outside the home |
| 87 | Sudden changes in mood or feelings | 90 | Swearing or obscene language |
| 88 | Sulks a lot | 96 | Thinks about sex too much |
| 89 | Suspicious | 99 | Smokes, chews, sniffs tobacco |
| 94 | Teases a lot | 101 | Truancy, skips school |
| 95 | Temper tantrums or hot temper | 105 | Uses drugs for nonmedical purposes |
| 97 | Threatens people | 106 | Vandalism |
| 104 | Unusually loud |  |  |


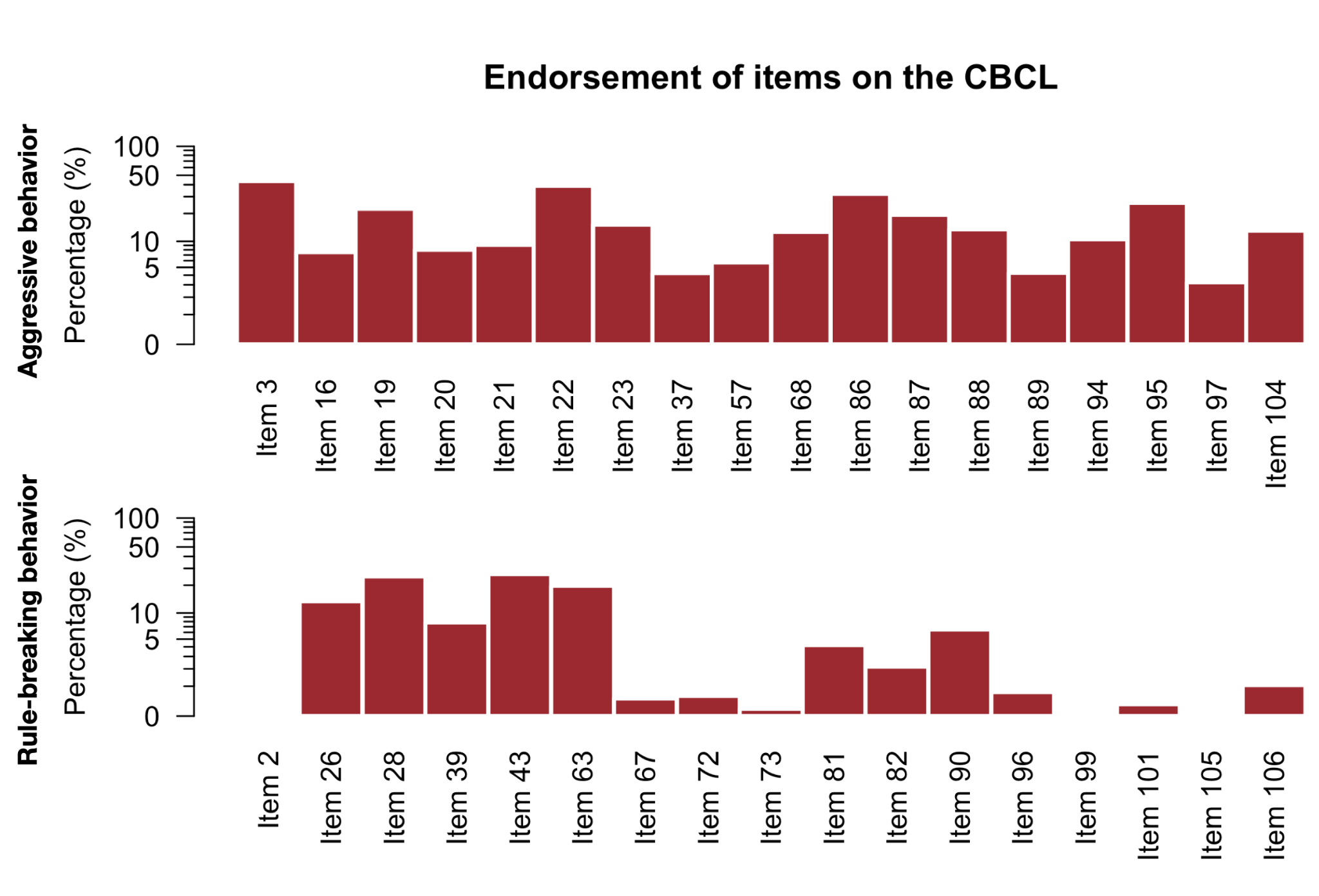


**Supplementary Figure S1**. Endorsement of individual items on the parent-reported Child Behavior Checklist. A response of ‘1: occasionally’ or ‘2: often’ was considered an endorsement. See **Supplementary Table S2** for description of items. Percentage of endorsement is plotted on a logarithmic scale for visualization purposes only. Abbreviation (in alphabetical order): CBCL = Child Behavior Checklist.

## Data transformations of summary scores

The distribution of the summary scores of externalizing behaviors were positively skewed toward the right due to a high number of zero responses from children without signs of disruptive behavior (**Supplementary Figure S2**). A log-normal distribution described the data better than a normal distribution, therefore, the raw summary scores were log_10_-transformed prior to statistical analysis.


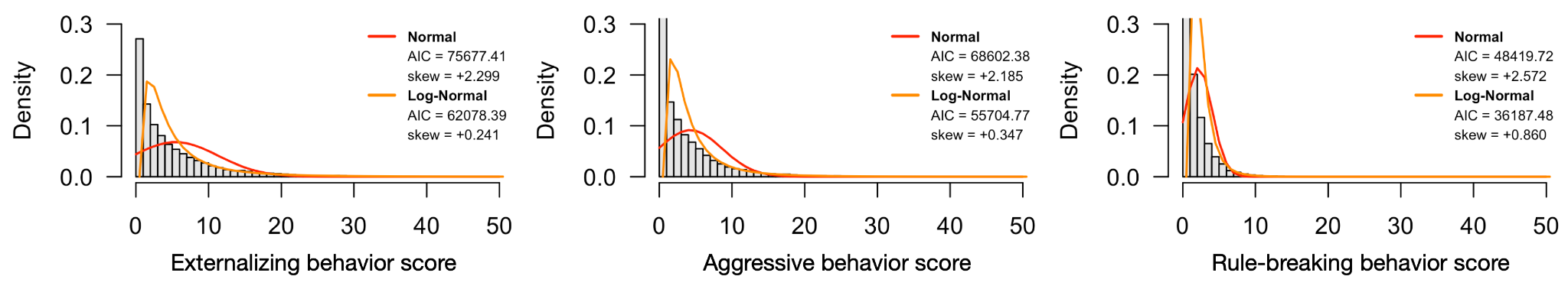


**Supplementary Figure S2.** Distribution of summary scores of externalizing, aggressive behavior, and rule-breaking behavior scales on the Child Behavior Checklist.

## Effect of covariates on externalizing behavior scores

The analyses of summary scores of the CBCL were corrected for sex, age, household income, parental education level, parental marital status, race/ethnicity of the child, and acquisition site (**Supplementary Table S3**).

**Supplementary Table S3.** Effect of covariates on parent-reported externalizing behaviors in the ABCD cohort.

| **Measure** | **Externalizing score** | **Aggressive score** | **Rule-breaking score** |
| --- | --- | --- | --- |
| Raw score ^a^ | 4.45±5.9 [0; 49] | 3.26±4.4 [0; 36] | 1.19±1.9 [0; 20] |
| Sex  “Female” vs “Male” | B=+0.253  ***p*=9.29E–34** | B=+0.194  ***p*=7.04E–25** | B=+0.186  ***p*=1.41E–43** |
| Age (months) | B=–0.0023  *p*=5.30E–02 | B=–0.0021  *p*=5.61E–02 | B=–0.0014  *p*=5.82E–02 |
| Household income ^b,c^  “<50K” vs “>=50K & <100K”  “<50k” vs “>= 100K”  Omnibus test | B=–0.098  ***p*=5.55E–04**  B=–0.178  ***p*=8.15E–09**  Δ–2LL=34.21  **p=3.72E–08** | B=–0.101  ***p*=1.71E–04**  B=–0.168  ***p*=6.71E–09**  Δ–2LL=34.14  **p=3.85E–08** | B=–0.067  ***p*=4.26E–04**  B=–0.115  ***p*=4.08E–08**  Δ–2LL=30.60  **p=2.27E–07** |
| Parental education ^b,c^  “<HS diploma/GED” vs “HS diploma/GED”  “<HS diploma/GED” vs “Some college degree”  “<HS diploma/GED” vs “Bachelor degree”  “<HS diploma/GED” vs “Post graduate degree”  Omnibus test | B=–0.090  *p*=1.03E–01  B=–0.023  *p*=6.58E–01  B=–0.098  *p*=6.67E–02  B=–0.155  ***p*=3.18E–03**  Δ–2LL=26.23  **p=2.85E–05** | B=–0.092  *p*=7.56E–02  B=–0.036  *p*=4.62E–01  B=–0.086  *p*=9.01E–02  B=–0.127  ***p*=1.14–02**  Δ–2LL=15.65  **p=3.52E–03** | B=–0.036  *p*=3.43E–01  B=+0.005  *p*=8.71E–01  B=–0.075  *p*=3.59E–02  B=–0.129  ***p*=2.26–04**  Δ–2LL=55.11  **p=3.08E–11** |
| Parental marital status ^c^  “Separated” vs “Married or living together” | B=–0.119  ***p*=8.89E–07** | B=–0.094  ***p*=4.33E–05** | B=–0.098  ***p*=6.12E–10** |
| Race/ethnicity ^b^  “White” vs “Black”  “White” vs “Hispanic”  “White” vs “Asian”  “White” vs “Other”  Omnibus test | B=–0.092  ***p*=4.70E–03**  B=–0.088  ***p*=3.35E–03**  B=–0.271  ***p*=4.69E–06**  B=+0.033  *p*=3.21E–01  Δ–2LL=36.28  **p=2.53E–07** | B=–0.129  ***p*=1.41E–03**  B=–0.084  ***p*=2.51E–03**  B=–0.276  ***p*=4.76E–07**  B=+0.002  *p*=9.33E–01  Δ–2LL=46.45  **p=1.98E–09** | B=+0.038  *p*=1.06E–01  B=–0.060  ***p*=1.93E–03**  B=–0.115  ***p*=6.20E–03**  B=+0.079  ***p*=5.03E–04**  Δ–2LL=40.60  **p=3.25E–08** |
| Acquisition site ^b,d^  Omnibus test | –2LL: 156.46  ***p*=1.03E–22** | –2LL: 145.80  ***p*=1.10E–20** | –2LL: 109.66  ***p*=5.43E–14** |
| Total variance explained (R^2^) | 5.87% | 4.78% | 7.70% |

*The reported values for covariates are the regression coefficient (after conversion back to linear scale), or difference in log-likelihood ratio between the null and restricted model for the omnibus tests, followed by the uncorrected p-value. P-values printed in* ***boldface*** *are significant at p_unc_ < 0.05.
^a^ The mean±SD and range [min; max] are reported for the untransformed raw scores on the CBCL scales.
^b^ Note that significance for the omnibus tests of household income, parental education, race/ethnicity, and acquisition site have respectively 2, 4, 4, and 21 degrees of freedom.*^c^ *Note that parental education, household income, and parental marital status are strongly collinear (polychoric correlation ranges between 0.47 to 0.74).*^c^ *Note that acquisition site is considered a covariate of no interest, but its effect may also represent geographic location*

## Brain structures

### Gray matter structures

The selection of cortical regions and subcortical structures was based on a previous study that investigated the association between gray matter volume and childhood disruptive behavior in the ABCD cohort [(Teeuw et al., 2022; Waller et al., 2020)](https://paperpile.com/c/enbthS/L1eP+HBWX) (**Supplementary Table S4**). In addition, the following global brain measures were extracted: total brain volume (TBV), total cortical gray matter volume (CGM), total subcortical gray matter volume (SCGM), and total cerebral white matter volume (CWM).

**Supplementary Table S4.** List of cortical and subcortical regions.

| **ID** | **Abbreviation** | **Full name** | **Cortex** |
| --- | --- | --- | --- |
| 1/2 | cACC | Caudal anterior cingulate gyrus | Cingulum |
| 3/4 | rACC | Rostral anterior cingulate gyrus | Cingulum |
| 5/6 | FG | Fusiform gyrus | Temporal |
| 7/8 | IFG | Inferior frontal gyrus | Frontal |
| 9/10 | Ins | Insular cortex | Insula |
| 11/12 | lOFG | Lateral orbitofrontal gyrus | Frontal |
| 13/14 | SFG | Superior frontal gyrus | Frontal |
| 15/16 | STG | Superior temporal gyrus | Temporal |
| 17/18 | mOFG | Medial orbitofrontal gyrus | Frontal |
| 19/20 | rMFG | Rostral middle frontal gyrus | Frontal |
| 21/22 | AMY | Amygdala | Subcortical |
| 23/24 | HIP | Hippocampus | Subcortical |
| 25 | TBV | Total brain volume | Global |
| 26 | CGM | Cortical gray matter volume | Global |
| 27 | SCGM | Subcortical gray matter volume | Global |
| 28 | CWM | Cerebral white matter volume | Global |

*Note that cortical regions and subcortical structures with two IDs are bilateral and have a separate left and right volume.*

### White matter tracts

The *AtlasTrack* software was used to automatically segment 37 major white matter tracts of the brain [(Hagler et al., 2019, 2009)](https://paperpile.com/c/enbthS/OELj+XHof). Here, we selected 20 of the major white matter tracts also represented by the John Hopkins University atlas, and in addition the corpus callosum and fornix, for comparison to other developmental studies (**Supplementary Table S5**). In addition, a global brain measure was defined by the voxels of all tracts combined. Fractional anisotropy (FA), mean diffusivity (MD), and volume (wmVol) were extracted for all tracts and global measures for a total of 72 measures of white matter microstructural integrity. Note that volume of white matter tracts were not available for the TwaalfMin cohort.

**Supplementary Table S5.** List of white matter tracts.

| **ID** | **Abbreviation** | **Full name** |
| --- | --- | --- |
| 1/2 | ATR | Anterior thalamic radiation |
| 3 | CC | Corpus callosum |
| 4/5 | CGC | Cingulum cingulate part |
| 6/7 | CGH | Cingulum hippocampal part |
| 8/9 | CST | Corticospinal tract |
| 10 | FMAJ | Forceps major |
| 11 | FMIN | Forceps minor |
| 12/13 | FX | Fornix |
| 14/15 | IFO | Inferior fronto-occipital fasciculus |
| 16/17 | ILF | Inferior longitudinal fasciculus |
| 18/19 | pSLF | Parietal superior longitudinal fasciculus |
| 20/21 | tSLF | Temporal superior longitudinal fasciculus |
| 22/23 | UNC | Uncinate fasciculus |
| 24 | AllTracts | All tracts combined |

*Note that white matter tracts with two IDs are bilateral and have a separate left and right white matter integrity measure.*

## Associations between externalizing PRS and externalizing behavior measures

Scores on the two CBCL subscales, i.e. aggressive (Agg-CBCL) and rule-breaking (Rb-CBCL) behaviors, were significantly associated with the PRS based on all three externalizing phenotypes GWAS (**Supplementary Table S6**).

**Supplementary Table S6.** Associations between polygenic risk scores (PRS) based on for externalizing phenotypes and CBCL aggressive and rule-breaking subscale scores in children from the general population.

| **PRS-base GWAS** | **Assoc. pheno in ABCD cohort** | **PRS P-threshold** | **PRS.R2** | **N SNPs** | **P** | **Empirical P** |
| --- | --- | --- | --- | --- | --- | --- |
| ADHD+DBD | CBCL-Agg | 0.1 | 0.0044 | 35664 | **1.036E-05** | **0.0002** |
|  | CBCL-Rb | 1 | 0.0019 | 138825 | **0.0037** | **0.0134** |
| ASB | CBCL-Agg | 0.2 | 0.00196 | 95045 | **0.0033** | **0.0105** |
|  | CBCL-Rb | 0.2 | 0.0029 | 95045 | **0.0003** | **0.0013** |
| Irritability | CBCL-Agg | 0.001 | 0.0040 | 3245 | **2.399E-05** | **9.999E-05** |
|  | CBCL-Rb | 0.3 | 0.0024 | 133697 | **0.0009** | **0.0041** |
| Traits related to self-regulation and addiction | CBCL-Agg | 0.1 | 0.0072 | 47059 | **4.325E-08** | **9.999E-05** |
|  | CBCL-Rb | 0.1 | 0.0119 | 47059 | **1.654E-12** | **9.999E-05** |

*P-values printed in* ***boldface*** *are significant at p_unc_ < 0.05. Abbreviations (in alphabetical order): ADHD+DBD =* attention-deficit/hyperactivity disorder comorbid with disruptive behavior disorders*; ASB = Antisocial behaviors; CBCL-Agg = aggressive behavior scale on the Child Behavior Checklist; CBCL-Rb = rule-breaking behavior scale on the Child Behavior Checklist; PRS = polygenic risk score; SNP = single nucleotide polymorphism.*

## Multivariate additive effect of externalizing PRS and brain structures

We employed multivariate regression models to investigate the combined effect of brain structures and polygenic risk scores on externalizing behavior. The variance explained by the predictors of the model were compared for different sets of brain structures, with or without the four polygenic risk scores (**Supplementary Table S7**).

**Supplementary Table S7.** Variance explained by multivariate models of brain structures and polygenic risk scores on externalizing behavior.

| **Model** | **Only brain structures** | | **Including PRSs** | |
| --- | --- | --- | --- | --- |
|  | **R^2^** | **ΔR^2^** | **R^2^** | **ΔR^2^** |
| Null (only sociodemographic factors and covariates) | 6.95% | N/A | 8.39% | +1,44% |
| Global brain measures | 7.64% | +0.69% | 9.06% | +2,11% |
| Regional (sub)cortical gray matter brain volumes | 8.14% | +1.19% | 9.51% | +2.56% |
| Regional fractional anisotropy (FA) of white matter tracts | 7.46% | +0.51% | 8.92% | +1.97% |
| Regional mean diffusivity (MD) of white matter tracts | 7.79% | +0.84% | 9.14% | +2.19% |
| Regional volume (wmVol) of white matter tracts | 7.65% | +0.70% | 9.10% | +2.15% |
| Regional diffusion measures of white matter tracts | 9.16% | +2.21% | 10.62% | +3.67% |
| All regional gray matter volumes and diffusion measures of white matter tracts | 10.13% | +3.18% | 11.50% | +4.55% |
| All global and regional brain measures | 10.54% | +3.59% | 11.90% | +4.95% |

Note that ΔR^2^ represents the difference in variance explained by the predictors (sociodemographic factors, brain structures and/or PRS) on externalizing behavior (CBCL-Ext) between the model with additional predictors and the Null model (without PRS or brain structures).

## Supplementary data files

**Supplementary Data File F1** is an Microsoft Excel spreadsheet that contains five worksheets with summary statistics and parameters from the regression models.

**Supplementary Data File F2** is a plain text file that contains the full output of the PROCESS macro in R for the mediation analysis of the global brain structures on the association between PRS and CBCL-Ext.
